# Quinoline-8-sulfonamide^1^

**DOI:** 10.1107/S1600536812036963

**Published:** 2012-09-01

**Authors:** Krzysztof Marciniec, Andrzej Maślankiewicz, Maria Nowak, Joachim Kusz

**Affiliations:** a Department of Organic Chemistry, The Medical University of Silesia, Jagiellońska 4, 41-200 Sosnowiec, Poland; b Institute of Physics, University of Silesia, Uniwersytecka 4, 40-007 Katowice, Poland

## Abstract

In the title compound, C_9_H_8_N_2_O_2_S, the sulfamoyl NH_2_ group is involved in intra­molecular N—H⋯N and inter­molecular N—H⋯O hydrogen bonding. In the crystal, molecules are linked *via* pairs of N—H⋯O hydrogen bonds, forming inversion dimers, which are further associated through π–π stacking inter­actions between the quinoline benzene rings [centroid–centroid distance = 3.649 (1) Å] into a one-dimensional polymeric structure extending along the *a* axis.

## Related literature

For the use of the quinoline­sulfamoyl unit in medicinal chemistry, see: Borras *et al.* (1999 ▶); Eveloch *et al.* (1981 ▶); Zajdel *et al.* (2011 ▶, 2012 ▶). For the synthesis, see: Maślankiewicz *et al.* (2007 ▶). For hydrogen-bonding motifs in sufonamides, see: Adsmond & Grant (2001 ▶). For graph-set notation of hydrgen-bond motifs, see: Bernstein *et al.* (1995 ▶).
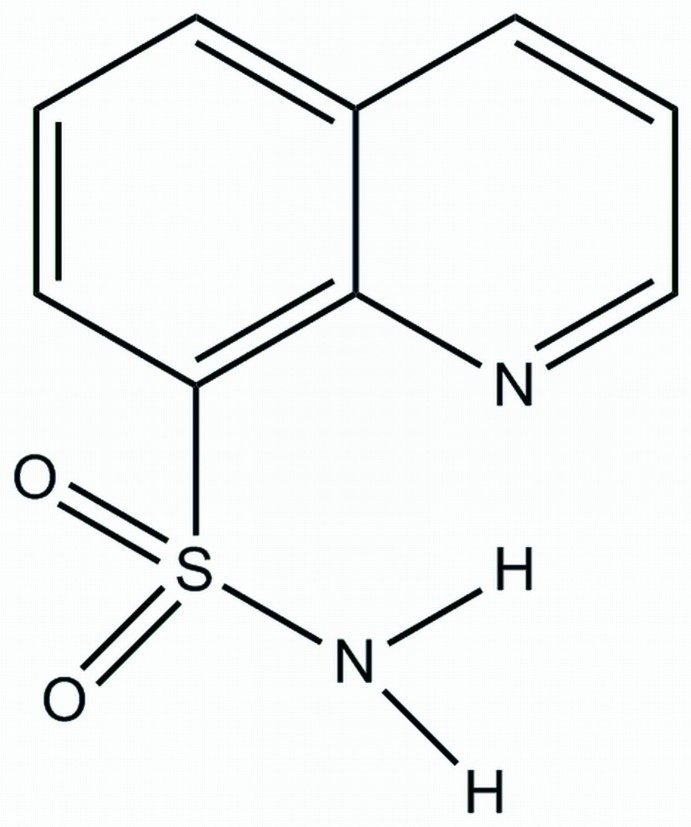


## Experimental

### 

#### Crystal data

C_9_H_8_N_2_O_2_S
*M* *_r_* = 208.23
Monoclinic, 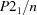

*a* = 8.9431 (3) Å
*b* = 10.4542 (2) Å
*c* = 10.4648 (2) Å
β = 109.313 (2)°
*V* = 923.33 (4) Å^3^
*Z* = 4
Mo *K*α radiation
μ = 0.32 mm^−1^
*T* = 298 K
0.34 × 0.21 × 0.18 mm

#### Data collection

Oxford Diffraction Xcalibur Sapphire3 CCD diffractometer
Absorption correction: multi-scan (*CrysAlis RED*; Oxford Diffraction, 2008 ▶) *T*_min_ = 0.898, *T*_max_ = 0.944
5936 measured reflections
1636 independent reflections
1446 reflections with *I* > 2σ(*I*)
*R*_int_ = 0.014

#### Refinement

*R*[*F* ^2^ > 2σ(*F* ^2^)] = 0.029
*wR*(*F* ^2^) = 0.095
*S* = 0.97
1636 reflections
135 parameters
H atoms treated by a mixture of independent and constrained refinement
Δρ_max_ = 0.31 e Å^−3^
Δρ_min_ = −0.36 e Å^−3^

### 

Data collection: *CrysAlis CCD* (Oxford Diffraction, 2008 ▶); cell refinement: *CrysAlis CCD*; data reduction: *CrysAlis RED* (Oxford Diffraction, 2008 ▶); program(s) used to solve structure: *SHELXS97* (Sheldrick, 2008 ▶); program(s) used to refine structure: *SHELXL97* (Sheldrick, 2008 ▶); molecular graphics: *Jmol* (Hanson, 2010 ▶) and *Mercury* (Macrae *et al.*, 2006 ▶); software used to prepare material for publication: *SHELXL97*.

## Supplementary Material

Crystal structure: contains datablock(s) I, global. DOI: 10.1107/S1600536812036963/gk2515sup1.cif

Supplementary material file. DOI: 10.1107/S1600536812036963/gk2515Isup2.mol

Structure factors: contains datablock(s) I. DOI: 10.1107/S1600536812036963/gk2515Isup3.hkl

Supplementary material file. DOI: 10.1107/S1600536812036963/gk2515Isup4.cml

Additional supplementary materials:  crystallographic information; 3D view; checkCIF report

## Figures and Tables

**Table 1:** Hydrogen-bond geometry (Å, °)

*D*—H⋯*A*	*D*—H	H⋯*A*	*D*⋯*A*	*D*—H⋯*A*
N2—H1*N*⋯O2^i^	0.87 (2)	2.15 (3)	3.013 (2)	169 (2)
N2—H2*N*⋯N1	0.83 (2)	2.33 (2)	2.921 (2)	129 (2)

Symmetry code: (i) 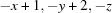.

**Fig. 1.**
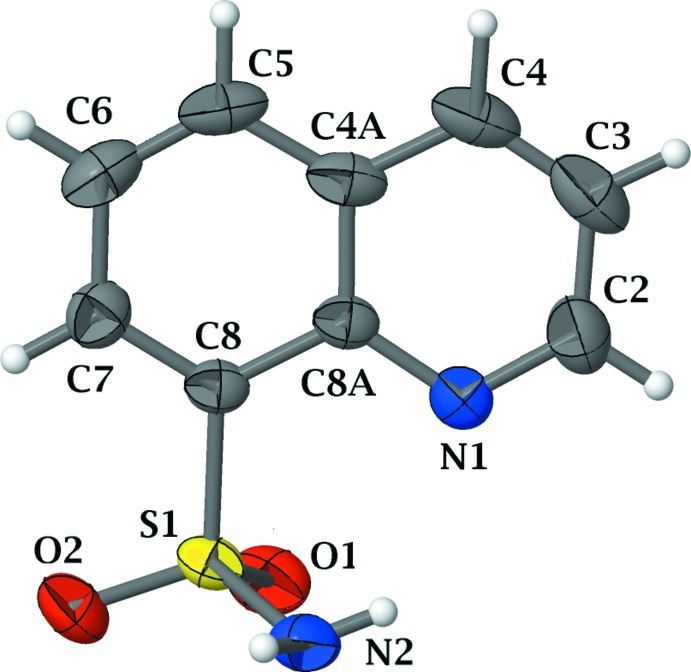
Molecular structure of the title compound with displacement ellipsoids drawn at the 50% probability level.

**Fig. 2.**
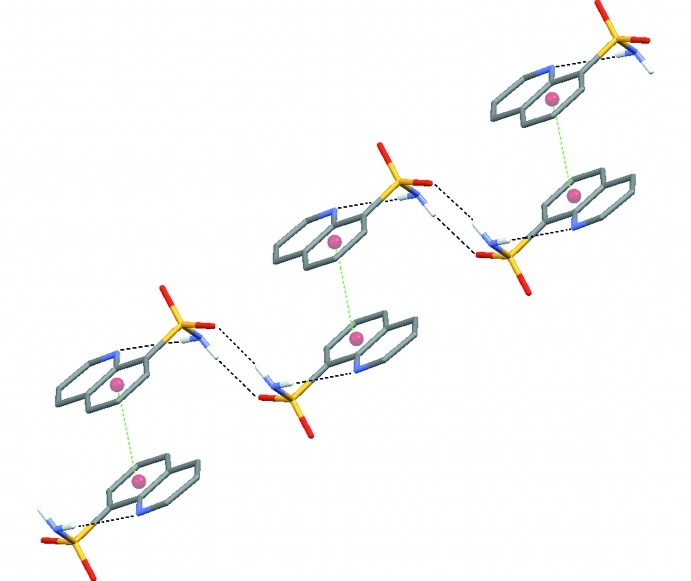
π-π stacking interactions (green dashed line) and hydrogen bonds (black dashed lines) in the title crystal structure. H atoms not involved in hydrogen bonding have been omitted for clarity.

**Fig. 3.**
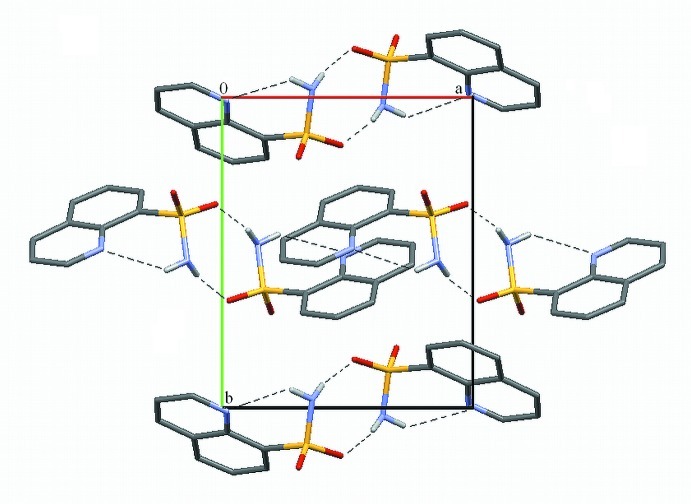
Crystal packing of the title compound along the c axis. Hydrogen bonds are shown as dashed lines. H atoms not involved in hydrogen bonding have been omitted for clarity.

## supplementary crystallographic information

### Comment

Quinolinesulfamoyl moiety is being more and more frequently incorporated into molecules of biologically active compounds such as carbonic anhydrase inhibitors (Borras *et al.*, 1999) and 5-HT receptors ligands (Zajdel *et al.*, 2011; Zajdel *et al.*, 2012). Since the quinoline drugs as well as sulfonamides strongly interact with enzymatic receptors *via* their nitrogen atoms (Eveloch *et al.*, 1981) we studied the crystal structure of the title compound, to evaluate the spatial environment of the nitrogen atoms.

The molecular conformation of quinoline-8-sulfonamide with the adopted atomic numbering is presented in Fig.1. The sulfonamide group participates in both intra- and intermolecular hydrogen bonding. The H2 atom of the sulfamoyl group shows an intramolecular contact with the N1 atom of the quinoline ring system (Table 1) resulting in the graph-set motif of *S*(6) (Bernstein *et al.*, 1995). In the crystal, the molecules form dimers through N2—H1···O2 hydrogen bonds (Table 1). It is interesting to note that the most commonly observed hydrogen bonding in sulfonamides in the studies reported by Adsmond &Grant (2001) consing of S=O···H—N chains (50 occurrences in 39 different sulfonamide structures) is absent in the title compound.

A π-π stacking interaction is observed between the benzene C4A/C5—C8/C8A rings of neighboring dimers with the centroid-to-centroid distance, *Cg*···*Cg* (1 - *x*, 2 - *y*, -*z*) of 3.649 (1) Å and interplanar spacing of 3.373 (1) Å (Fig. 2). The π–π stacking interaction connects the dimers along the [100] direction forming one-dimesional polymeric structure.

### Experimental

The title compound was prepared by the reaction of 8-quinolinesulfonylchloride with an excess ammonia at temperature of 45°C according to the procedure reported by Maślankiewicz *et al.* (2007). Single crystals of the title compound suitable for X-ray structure determination were obtained by recrystallization from an ethanolic solution.

### Refinement

The hydrogen atoms participating in hydrogen bonding were located in a difference Fourier map and freely refined. Other hydrogen atoms were introduced in geometrically idealized positions and refined using a riding-model approximation with C—H distances of 0.93 Å and with *U*_iso_(H)= 1.2*U*_eq_(C).

### Crystal data

**Table:**

C_9_H_8_N_2_O_2_S	*F*(000) = 432
*M**_r_* = 208.23	*D*_x_ = 1.498 Mg m^−^^3^
Monoclinic, *P*2_1_/*n*	Melting point: 457.2 K
Hall symbol: -P 2yn	Mo *K*α radiation, λ = 0.71073 Å
*a* = 8.9431 (3) Å	Cell parameters from 5251 reflections
*b* = 10.4542 (2) Å	θ = 3.1–34.5°
*c* = 10.4648 (2) Å	µ = 0.32 mm^−^^1^
β = 109.313 (2)°	*T* = 298 K
*V* = 923.33 (4) Å^3^	Polyhedron, colourless
*Z* = 4	0.34 × 0.21 × 0.18 mm

### Data collection

**Table:**

Oxford Diffraction Xcalibur Sapphire3 CCD diffractometer	1636 independent reflections
Radiation source: fine-focus sealed tube	1446 reflections with *I* > 2σ(*I*)
Graphite monochromator	*R*_int_ = 0.014
Detector resolution: 16.0328 pixels mm^-1^	θ_max_ = 25.1°, θ_min_ = 3.1°
ω–scan	*h* = −8→10
Absorption correction: multi-scan (*CrysAlis RED*; Oxford Diffraction, 2008)	*k* = −12→11
*T*_min_ = 0.898, *T*_max_ = 0.944	*l* = −12→10
5936 measured reflections	

### Refinement

**Table:**

Refinement on *F*^2^	Primary atom site location: structure-invariant direct methods
Least-squares matrix: full	Secondary atom site location: difference Fourier map
*R*[*F*^2^ > 2σ(*F*^2^)] = 0.029	Hydrogen site location: inferred from neighbouring sites
*wR*(*F*^2^) = 0.095	H atoms treated by a mixture of independent and constrained refinement
*S* = 0.97	*w* = 1/[σ^2^(*F*_o_^2^) + (0.075*P*)^2^ + 0.1368*P*] where *P* = (*F*_o_^2^ + 2*F*_c_^2^)/3
1636 reflections	(Δ/σ)_max_ = 0.001
135 parameters	Δρ_max_ = 0.31 e Å^−^^3^
0 restraints	Δρ_min_ = −0.36 e Å^−^^3^

### Special details

**Table:**

Geometry. All e.s.d.'s (except the e.s.d. in the dihedral angle between two l.s. planes) are estimated using the full covariance matrix. The cell e.s.d.'s are taken into account individually in the estimation of e.s.d.'s in distances, angles and torsion angles; correlations between e.s.d.'s in cell parameters are only used when they are defined by crystal symmetry. An approximate (isotropic) treatment of cell e.s.d.'s is used for estimating e.s.d.'s involving l.s. planes.
Refinement. Refinement of *F*^2^ against ALL reflections. The weighted *R*-factor *wR* and goodness of fit *S* are based on *F*^2^, conventional *R*-factors *R* are based on *F*, with *F* set to zero for negative *F*^2^. The threshold expression of *F*^2^ > σ(*F*^2^) is used only for calculating *R*-factors(gt) *etc*. and is not relevant to the choice of reflections for refinement. *R*-factors based on *F*^2^ are statistically about twice as large as those based on *F*, and *R*- factors based on ALL data will be even larger.

### Fractional atomic coordinates and isotropic or equivalent isotropic displacement parameters (Å^2^)

**Table:**

	*x*	*y*	*z*	*U*_iso_*/*U*_eq_	
S1	0.66396 (4)	0.87959 (4)	0.17318 (4)	0.03962 (18)	
O1	0.69562 (15)	0.80754 (13)	0.29483 (13)	0.0560 (4)	
O2	0.52811 (14)	0.84522 (13)	0.05984 (13)	0.0542 (4)	
N1	0.98319 (16)	0.98818 (14)	0.31765 (14)	0.0438 (3)	
N2	0.6409 (2)	1.02696 (16)	0.2070 (2)	0.0501 (4)	
C2	1.1202 (2)	1.03811 (18)	0.3911 (2)	0.0556 (5)	
H2	1.1242	1.0848	0.4678	0.067*	
C3	1.2598 (2)	1.0245 (2)	0.3596 (2)	0.0637 (6)	
H3	1.3541	1.0596	0.4160	0.076*	
C4	1.2566 (2)	0.96016 (18)	0.2470 (2)	0.0568 (5)	
H4	1.3485	0.9520	0.2245	0.068*	
C4A	1.1134 (2)	0.90515 (16)	0.16339 (19)	0.0430 (4)	
C5	1.0985 (2)	0.83950 (17)	0.0427 (2)	0.0513 (5)	
H5	1.1871	0.8287	0.0161	0.062*	
C6	0.9568 (2)	0.79162 (18)	−0.0357 (2)	0.0540 (5)	
H6	0.9487	0.7495	−0.1161	0.065*	
C7	0.8221 (2)	0.80560 (16)	0.00428 (17)	0.0448 (4)	
H7	0.7254	0.7722	−0.0494	0.054*	
C8	0.83294 (17)	0.86792 (14)	0.12156 (15)	0.0341 (4)	
C8A	0.97904 (17)	0.92153 (14)	0.20522 (15)	0.0351 (3)	
H1N	0.603 (3)	1.071 (2)	0.133 (3)	0.059 (6)*	
H2N	0.722 (3)	1.057 (2)	0.263 (3)	0.065 (7)*	

### Atomic displacement parameters (Å^2^)

**Table:**

	*U*^11^	*U*^22^	*U*^33^	*U*^12^	*U*^13^	*U*^23^
S1	0.0313 (3)	0.0447 (3)	0.0465 (3)	−0.00383 (16)	0.01779 (19)	0.00320 (17)
O1	0.0532 (8)	0.0673 (9)	0.0553 (8)	−0.0052 (6)	0.0284 (6)	0.0151 (6)
O2	0.0352 (6)	0.0582 (8)	0.0648 (8)	−0.0102 (5)	0.0106 (6)	−0.0017 (6)
N1	0.0371 (7)	0.0500 (8)	0.0436 (8)	−0.0022 (6)	0.0122 (6)	−0.0040 (6)
N2	0.0378 (8)	0.0549 (10)	0.0613 (10)	0.0023 (7)	0.0215 (8)	−0.0066 (9)
C2	0.0484 (10)	0.0542 (11)	0.0545 (11)	−0.0042 (8)	0.0039 (8)	−0.0058 (8)
C3	0.0357 (10)	0.0557 (12)	0.0876 (16)	−0.0070 (8)	0.0040 (10)	0.0055 (11)
C4	0.0326 (8)	0.0488 (10)	0.0919 (15)	0.0036 (7)	0.0247 (9)	0.0119 (10)
C4A	0.0367 (9)	0.0358 (8)	0.0629 (11)	0.0066 (7)	0.0250 (8)	0.0120 (7)
C5	0.0562 (11)	0.0431 (9)	0.0718 (12)	0.0111 (8)	0.0443 (10)	0.0076 (9)
C6	0.0732 (13)	0.0457 (10)	0.0560 (10)	0.0069 (9)	0.0389 (10)	−0.0030 (8)
C7	0.0509 (10)	0.0404 (9)	0.0448 (9)	−0.0021 (7)	0.0181 (8)	−0.0018 (7)
C8	0.0337 (8)	0.0322 (8)	0.0396 (8)	0.0008 (6)	0.0166 (6)	0.0045 (6)
C8A	0.0333 (8)	0.0328 (8)	0.0418 (8)	0.0017 (6)	0.0161 (7)	0.0047 (6)

### Geometric parameters (Å, º)

**Table:**

S1—O1	1.4247 (13)	C4—C4A	1.413 (3)
S1—O2	1.4348 (12)	C4—H4	0.9300
S1—N2	1.6092 (17)	C4A—C5	1.405 (3)
S1—C8	1.7693 (15)	C4A—C8A	1.419 (2)
N1—C2	1.320 (2)	C5—C6	1.357 (3)
N1—C8A	1.357 (2)	C5—H5	0.9300
N2—H1N	0.87 (2)	C6—C7	1.407 (2)
N2—H2N	0.83 (2)	C6—H6	0.9300
C2—C3	1.400 (3)	C7—C8	1.364 (2)
C2—H2	0.9300	C7—H7	0.9300
C3—C4	1.349 (3)	C8—C8A	1.425 (2)
C3—H3	0.9300		
			
O1—S1—O2	118.03 (8)	C5—C4A—C4	123.55 (17)
O1—S1—N2	108.14 (10)	C5—C4A—C8A	119.89 (16)
O2—S1—N2	106.66 (9)	C4—C4A—C8A	116.54 (17)
O1—S1—C8	107.39 (7)	C6—C5—C4A	121.06 (15)
O2—S1—C8	107.79 (8)	C6—C5—H5	119.5
N2—S1—C8	108.52 (7)	C4A—C5—H5	119.5
C2—N1—C8A	117.50 (15)	C5—C6—C7	120.09 (16)
S1—N2—H1N	110.7 (15)	C5—C6—H6	120.0
S1—N2—H2N	112.0 (16)	C7—C6—H6	120.0
H1N—N2—H2N	115 (2)	C8—C7—C6	120.29 (16)
N1—C2—C3	123.47 (19)	C8—C7—H7	119.9
N1—C2—H2	118.3	C6—C7—H7	119.9
C3—C2—H2	118.3	C7—C8—C8A	121.20 (14)
C4—C3—C2	119.58 (17)	C7—C8—S1	119.61 (12)
C4—C3—H3	120.2	C8A—C8—S1	119.17 (12)
C2—C3—H3	120.2	N1—C8A—C4A	123.12 (15)
C3—C4—C4A	119.77 (17)	N1—C8A—C8	119.41 (13)
C3—C4—H4	120.1	C4A—C8A—C8	117.45 (15)
C4A—C4—H4	120.1		
			
C8A—N1—C2—C3	−0.7 (3)	O1—S1—C8—C8A	−64.59 (13)
N1—C2—C3—C4	1.7 (3)	O2—S1—C8—C8A	167.26 (12)
C2—C3—C4—C4A	−1.2 (3)	N2—S1—C8—C8A	52.10 (15)
C3—C4—C4A—C5	178.30 (18)	C2—N1—C8A—C4A	−0.8 (2)
C3—C4—C4A—C8A	−0.2 (3)	C2—N1—C8A—C8	−178.87 (15)
C4—C4A—C5—C6	−178.12 (17)	C5—C4A—C8A—N1	−177.31 (15)
C8A—C4A—C5—C6	0.4 (3)	C4—C4A—C8A—N1	1.3 (2)
C4A—C5—C6—C7	−1.0 (3)	C5—C4A—C8A—C8	0.8 (2)
C5—C6—C7—C8	0.4 (3)	C4—C4A—C8A—C8	179.37 (14)
C6—C7—C8—C8A	0.8 (2)	C7—C8—C8A—N1	176.81 (15)
C6—C7—C8—S1	−177.78 (13)	S1—C8—C8A—N1	−4.61 (19)
O1—S1—C8—C7	114.02 (14)	C7—C8—C8A—C4A	−1.4 (2)
O2—S1—C8—C7	−14.14 (15)	S1—C8—C8A—C4A	177.22 (11)
N2—S1—C8—C7	−129.30 (14)		

### Hydrogen-bond geometry (Å, º)

**Table:**

*D*—H···*A*	*D*—H	H···*A*	*D*···*A*	*D*—H···*A*
N2—H1*N*···O2^i^	0.87 (2)	2.15 (3)	3.013 (2)	169 (2)
N2—H2*N*···N1	0.83 (2)	2.33 (2)	2.921 (2)	129 (2)

Symmetry code: (i) −*x*+1, −*y*+2, −*z*.
